# Induction of a Torpor-Like State by 5’-AMP Does Not Depend on H_2_S Production

**DOI:** 10.1371/journal.pone.0136113

**Published:** 2015-08-21

**Authors:** George J. Dugbartey, Hjalmar R. Bouma, Arjen M. Strijkstra, Ate S. Boerema, Robert H. Henning

**Affiliations:** 1 Department of Clinical Pharmacy and Pharmacology, University of Groningen, University Medical Center Groningen, Groningen, The Netherlands; 2 Departments of Chronobiology and Molecular Neurobiology, Groningen Institute for Evolutionary Life Sciences, University of Groningen, 9700 RB, Groningen, The Netherlands; 3 Department of Nuclear Medicine and Molecular Imaging, University Medical Center Groningen, University of Groningen, Groningen, The Netherlands; University Medical Center Utrecht, NETHERLANDS

## Abstract

**Background:**

Therapeutic hypothermia is used to reduce ischemia/reperfusion injury (IRI) during organ transplantation and major surgery, but does not fully prevent organ injury. Interestingly, hibernating animals undergo repetitive periods of low body temperature called ‘torpor’ without signs of organ injury. Recently, we identified an essential role of hydrogen sulfide (H_2_S) in entrance into torpor and preservation of kidney integrity during hibernation. A torpor-like state can be induced pharmacologically by injecting 5’-Adenosine monophosphate (5’-AMP). The mechanism by which 5’-AMP leads to the induction of a torpor-like state, and the role of H_2_S herein, remains to be unraveled. Therefore, we investigated whether induction of a torpor-like state by 5-AMP depends on H_2_S production.

**Methods:**

To study the role of H_2_S on the induction of torpor, amino-oxyacetic acid (AOAA), a non-specific inhibitor of H_2_S, was administered before injection with 5'-AMP to block endogenous H_2_S production in Syrian hamster. To assess the role of H_2_S on maintenance of torpor induced by 5’-AMP, additional animals were injected with AOAA during torpor.

**Key Results:**

During the torpor-like state induced by 5’-AMP, the expression of H_2_S- synthesizing enzymes in the kidneys and plasma levels of H_2_S were increased. Blockade of these enzymes inhibited the rise in the plasma level of H_2_S, but neither precluded torpor nor induced arousal. Remarkably, blockade of endogenous H_2_S production was associated with increased renal injury.

**Conclusions:**

Induction of a torpor-like state by 5’-AMP does not depend on H_2_S, although production of H_2_S seems to attenuate renal injury. Unraveling the mechanisms by which 5’-AMP reduces the metabolism without organ injury may allow optimization of current strategies to limit (hypothermic) IRI and improve outcome following organ transplantation, major cardiac and brain surgery.

## Introduction

Therapeutic hypothermia is a commonly used technique to prevent ischemia/reperfusion injury (IRI) during major cardiac and neuronal surgery and following cardiopulmonary resuscitation. Although hypothermia reduces ischemia by lowering the metabolism, therapeutic hypothermia does not completely preclude organ injury. The generation of reactive oxygen species is the major culprit in IRI [1]. Interestingly, hibernating animals cycle through a state of lowered metabolism with a profoundly reduced body temperature called ‘torpor’ and periods of euthermia called ‘arousal’, without gross signs of organ injury [2–5]. The duration of a torpor bout depends on the species and varies from several days to a month. In hibernating arctic ground squirrels, for example, the body temperature during torpor may be reduced towards freezing point, and is typically close to the ambient temperature [3,6–10]. Recently, Blackstone *et al* [11] demonstrated that inhalation of H_2_S induced a hibernation-like state in mice for 6 hours followed by a full recovery without behavioral changes. Moreover, lung tissue H_2_S is increased during torpor in the Syrian hamster [7]. Plasma levels of acid-labile sulfur, which consists of Fe-S clusters that can be converted into H_2_S under acidic conditions, are increased during hibernation in the brown bear [12]. However, the plasma levels of bound sulfur, which can be converted into H_2_S under reducing conditions, and unbound sulfur, which consists of freely dissolved H_2_S and HS-, on the other hand, are reduced during hibernation in the brown bear. These specific alterations with regard to plasma sulfur suggest that in addition to increased production, also H_2_S consumption is changed during hibernation. Endogenous H_2_S can be produced by cystathionine-β-synthase (CBS), cystathionine-γ-lyase (CSE) and 3-mercaptopyruvate-sulfurtransferase (MST). Previously, we showed that during torpor in the Syrian hamster, CBS expression is increased in pulmonary tissue [7].

A torpor-like state can be induced pharmacologically in non-hibernating animals through inhalation of H_2_S or injection of 5’-adenosine monophosphate (5’-AMP), thereby mimicking natural torpor [13–16]. Fasting of mice housed under constant darkness, stimulates torpor behavior which is associated with increased levels of 5’-AMP in plasma [13], suggesting that 5’-AMP may be involved in the induction of natural torpor. Infusion of 5’-AMP activates the molecular energy sensor adenosine monophosphate kinase (AMPK), which mediated the protective effects of ischemic preconditioning on IRI [16]. Interestingly, H_2_S governs protection against lethal hypoxia in mice [16]. Infusion of 5’-AMP activates the molecular energy sensor adenosine monophosphate kinase (AMPK), which mediated the protective effects of ischemic preconditioning on IRI [17]. Further, infusion of 5’-AMP in rats limits activation of mitogen-activated protein kinases (MAP-kinases) and NFkB and pulmonary inflammation in models of endotoxemia [17–18]. The mechanisms underlying 5’-AMP mediated induction of a torpor-like state remain to be unraveled. Given the similarity of 5’-AMP and H_2_S on the induction of this torpor-like state and the preservation of organ integrity, we hypothesized that 5’-AMP may mediate its effects through stimulation of H_2_S production. To study whether the induction of a torpor-like state and preservation of kidney integrity by 5’-AMP depends on H_2_S, we measured the effect of 5’-AMP on activity, body temperature, kidney function and morphology in Syrian hamsters that were co-infused with either saline or the non-specific inhibitor of H_2_S production, amino-oxyacetic acid (AOAA). To exclude the influence of interspecies differences, we studied involvement of H_2_S in 5’-AMP induced torpor-like state and the prevention of kidney injury in a natural hibernator, i.e. Syrian hamster, the same species in which we revealed the essential role of H_2_S in the induction of natural torpor and reversible remodeling of lung tissue [7].

## Materials and Methods

### Ethical statement

All animal work has been conducted according to relevant national and international guidelines, and was approved by the Institutional Animal Ethical Committees of the University Medical Center Groningen.

### Experimental animals

Prior to experiments, male Syrian hamsters (*Mesocricetus auratus*) weighing 160g ±20 from Harlan Laboratories, Germany, were fed *ad libitum* using standard animal lab chow and animals were housed in groups of 4 animals per cage under normal light/dark conditions (L: D-cycle 12: 12 hours) at an ambient temperature of 20–25°C. Animals were randomly assigned to one of four groups, being control (n = 7), 5’-AMP with saline (n = 6), 5’-AMP with AOAA prior to torpor (n = 6; AOAA early) and 5’-AMP followed by AOAA during torpor (n = 6; AOAA late).

### Experiment procedures

Three weeks before the experiment, i-Button temperature loggers (Maxim Direct, France, DS1920 model) sealed in paraffin, were implanted intraperitoneally under isoflurane and analgesia with flunixin/meglumine (2 mg/kg). A blood sample (300 microliter) was obtained at baseline to measure sulfide levels and creatinine, as a measure of renal function. One day before start of the experiment, animals were housed individually in a climate-controlled room at 5°C. After 24 hours, animals were injected intraperitoneally with saline or AOAA (100 mg/kg) followed by 3 μmol/g 5'-AMP (3 mmol/kg, which equals about 1 mg/kg; in 0.9% saline, pH 7.5; Sigma Aldrich, The Netherlands) to induce a torpor-like state. Animals were euthanized by injecting an overdose of pentobarbital intraperitoneally 10 hours after injection of 5’-AMP. Next, a blood sample was drawn by cardiac puncture. Kidney samples were snap-frozen in liquid nitrogen and fixated in formaldehyde.

### (Immune)histochemistry

Kidney samples were fixated in 4% paraformaldehyde for 3 hours at room temperature followed by 4°C for 24 hours. Next, samples were dehydrated using a decreasing series of ethanol for 12 hours and embedded in paraffin. Four μm thick sections were deparaffinized in xylene (twice 5 minutes), followed by rehydration in a decreasing series of ethanol and distilled water. To evaluate changes in glomerular and tubular morphology, the kidney sections were stained with hematoxylin/eosin. Renal sections were examined blindly by two independent observers [19]. Glomerular damage was scored semiquantitativily in 100 glomeruli from 0 to 4 [20] and tubulointerstitial damage was quantified on the basis of tubular dilatation, atrophy of epithelial cells and widening of tubular lumen [19]. To evaluate the renal damage, sections were stained for kidney injury molecule (KIM-1, diluted 1: 50 v/v), a marker for renal tubular damage (Santa Cruz, The Netherlands), ED-1, a marker for macrophages (CD68, diluted 1: 500 v/v, Serotec Ltd, United Kingdom). Secondary and tertiary antibodies used are Horse Radish Peroxidase (HRP)-linked polyclonal rabbit anti-mouse IgG (diluted 1: 100 v/v), HRP-linked polyclonal rabbit anti-goat IgG (diluted 1: 100 v/v), and HRP-linked polyclonal goat anti-rabbit IgG (diluted 1: 100 v/v). Kidney sections were subjected to antigen retrieval in 0.1M Tris/HCl buffer (pH 9.0) by overnight incubation at 80°C. Next, sections were washed in PBS and blocked in 500 μl of 30% H_2_O_2_ for 30 minutes followed by incubation with the appropriate primary antibody for 60 minutes at room temperature. Following an additional washing step with PBS, samples were incubated for 30 minutes at room temperature with the appropriate secondary antibody and then with tertiary antibody at room temperature for 30 minutes. Finally, following a last washing step, samples were incubated with either DAB or AEC for 10–20 minutes and covered in either Depex mounting medium or DAKO Faramount aqueous mounting medium, and cover slips were applied.

### Western Blotting

Frozen kidney tissue samples (~500 mg) were homogenized in 400 μl RIPA buffer, consisting of 40 μl protease inhibitor cocktail (prepared according to the manufacturer’s instructions, Roche, The Netherlands), 2.5 mM sodium orthovanadate (Sigma Aldrich, The Netherlands) and 10 mM β-mercaptoethanol (Sigma Aldrich, The Netherlands). After 30 minutes incubation on ice, the homogenized samples were centrifuged at 14.000 g at 4°C for 20 minutes. Supernatants were collected and protein concentrations were determined using a Bradford protein assay, according to the manufacturer’s prescriptions (Bio-Rad, Germany). Samples were boiled for 5 minutes. SDS-polyacrylamide gel electrophoresis was run using 40 μg of protein per slot at 100V for 60 minutes. Proteins were then wet blotted onto nitrocellulose membranes (Bio-Rad, Germany) using a transfer buffer solution containing 0.25mM Tris (pH 8.5), 192 mM glycine and 10% v/v methanol at 4°C for 60 minutes at 0.3 mA. Next, the nitrocellulose membranes were blocked for 30 minutes in TBS + Tween-20 (50mM Tris-HCl, pH 6.8, 150mM NaCl, 0.05% v/v Tween-20) supplemented with 5% w/v skim milk. After decantation of the blocking buffer, membranes were incubated overnight at 4°C with the primary antibody diluted 1: 1000 v/v in 3% BSA/TBST (anti-CBS and anti-3-MST, Santa Cruz, The Netherlands; anti-CSE, Abnova, USA). Subsequently, membranes were washed three times in TBS buffer and incubated with HRP-linked polyclonal rabbit anti-goat IgG secondary antibody (1: 1000 v/v dilution) in TBS + Tween-20 supplemented with 3% BSA (w/v) for 60 minutes. Blots were developed using the SuperSignal West Dura Extended Duration Substrate (Thermo Scientific, USA) according to the manufacturer’s protocol. Protein bands were visualized using the Gene Genome system (Westburg B.V., The Netherlands) and band intensities were quantified using Gene Tools software (Westburg B.V., The Netherlands). β-actin was used as a house-keeping protein to normalize protein concentrations.

### Plasma H_2_S Measurement

Sulfide antioxidant buffer was prepared from 25 g of sodium salicylate, 6.5 g of ascorbic acid and 8.5 g of sodium hydroxide in 100 mL of distilled water and pH adjusted to ≥ 13. The sodium salicylate and ascorbic acid ensure that sulfide is in the form of sulfide ion (S^2-^). Next, 100 μL of the sulfide antioxidant buffer was added to 100 μL plasma samples. A sulfide ion sensitive electrode (Lazer Research Laboratories Inc., USA) was immersed into the mixture after 20 minutes [21] and the electrode potential was monitored and the stabilized mV reading was recorded. The S^2-^ concentration of the plasma was calculated using the electrode standardization curve prepared from 10 mL of the sulfide antioxidant buffer and 24 mg of Na_2_S.9H_2_O, according to the manufacturer’s guide.

### Statistical Analysis

All values are expressed as mean ± standard error of the mean (SEM). Differences between groups were tested for significance using a One-Way ANOVA. P-values < 0.05 were considered statistically significant. Significant differences were calculated with SPSS version 22 and graphs were produced using Sigmaplot version 13 for Windows.

## Results

### Endogenous H_2_S is not essential for the induction of a torpor-like state by 5’-AMP

**Fig 1:**
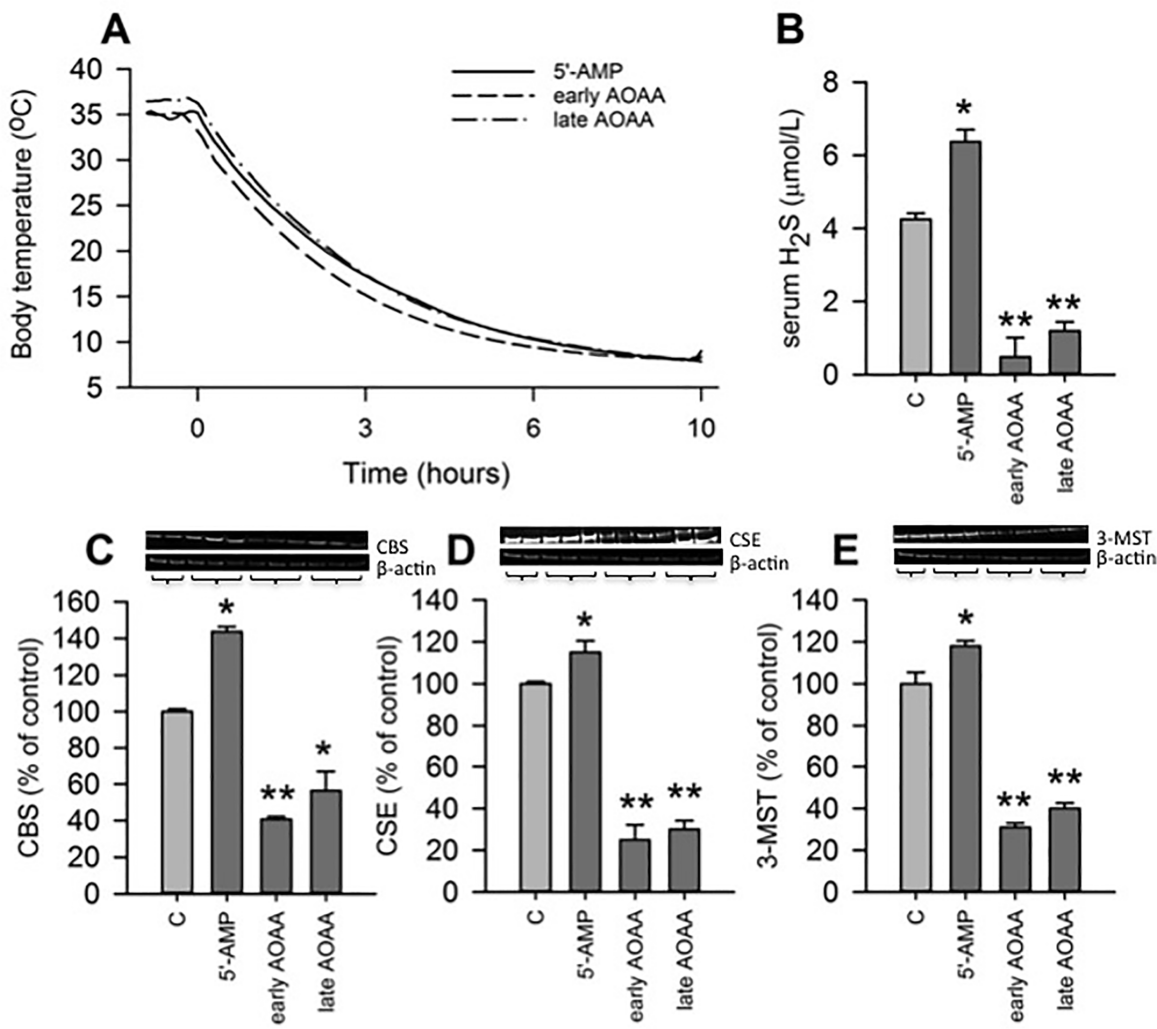
5’-AMP induces torpor in natural hibernators and increases plasma H_2_S and renal expression of H_2_S synthesizing enzymes. (A) Administration of 5’-AMP (at t = 0) resulted in a drop in core body temperature from 37°C to ~7°C in all three experimental groups after 10 h of 5’-AMP injection, which was not affected by early or late administration of AOAA. (B) Administration of 5’-AMP (at t = 0) significantly increased plasma H_2_S level compared to control animals, while AOAA injection prior to or 4 h after 5’-AMP administration reduced plasma H_2_S level. Also, AOAA administration reduced plasma H_2_S level in control animals. (C-E) Administration of 5’-AMP (at t = 0) upregulated the renal expression of CBS, CSE and 3-MST, while expression was decreased by AOAA injection prior to or 4 h after 5’-AMP administration. C = control animals */**, *p < 0*.*05/0*.*01* compared to control. Data are presented as mean ± SEM.

Injection of 5’-AMP in summer euthermic hamsters induced a torpor-like state as characterized by inactivity and marked drop in core body temperature from 37°C to 7°C, which lasted at least 10 hours (Fig 1A). At the time of euthanization, all animals were in the torpor-like state. To determine the involvement of H_2_S in 5’-AMP-induced torpor, we measured the plasma levels of H_2_S and blocked endogenous production either before or during torpor by AOAA. 5’-AMP-induced torpor significantly increased the endogenous H_2_S plasma level to ~150% of the plasma H_2_S level of summer euthermic control animals (Fig 1B; *p < 0*.*05*). Given that administration of exogenous H_2_S can induce a torpor-like state in mice [11], the increased plasma levels of H_2_S during torpor induced by 5’-AMP may suggest a role for H_2_S during torpor induced by 5’-AMP as well. Blocking endogenous H_2_S production by AOAA prior to 5’-AMP injection did not prevent the 5’-AMP-induced hypothermia (Fig 1A), although it substantially decreased plasma H_2_S level at 10 hours following injection of 5’-AMP to 11.4% of control animals (Fig 1B; *p < 0*.*01*). Further, injection of AOAA during the torpor-like state, 4 hours after injection of 5’-AMP, reduced the plasma level of H_2_S to 22.7% at 10 hours following injection of 5’-AMP (Fig 1B; *p < 0*.*01*). Despite the effect of AOAA on the plasma level of H_2_S, blockade of H_2_S production did not induce arousal. To determine whether the increased plasma level of H_2_S is due to changes in the amount of H_2_S-producing enzymes, we measured the amount of CBS, CSE and 3-MST in the kidney by Western Blot. As expected based on the effect of 5’-AMP on plasma H_2_S levels, administration of 5’-AMP resulted in a significant upregulation of all three H_2_S-producing enzymes, as compared to control animals (Fig 1C–1E; *p < 0*.*05*). Further, injection of AOAA, either prior to or during torpor, resulted in a significant lower amount of CBS, CSE and 3-MST as compared to control animals (Fig 1C–1E; *p < 0*.*05*). Thus, injection of 5’-AMP induces a torpor-like state in hamsters, which is not precluded by blocking H_2_S production, although 5’-AMP increases the plasma level of H_2_S, potentially due to an increased amount of CBS, CSE and 3-MST in kidneys and possibly in other organs.

### Blocking H_2_S production markedly increases plasma creatinine in 5’-AMP induced torpor

**Fig 2:**
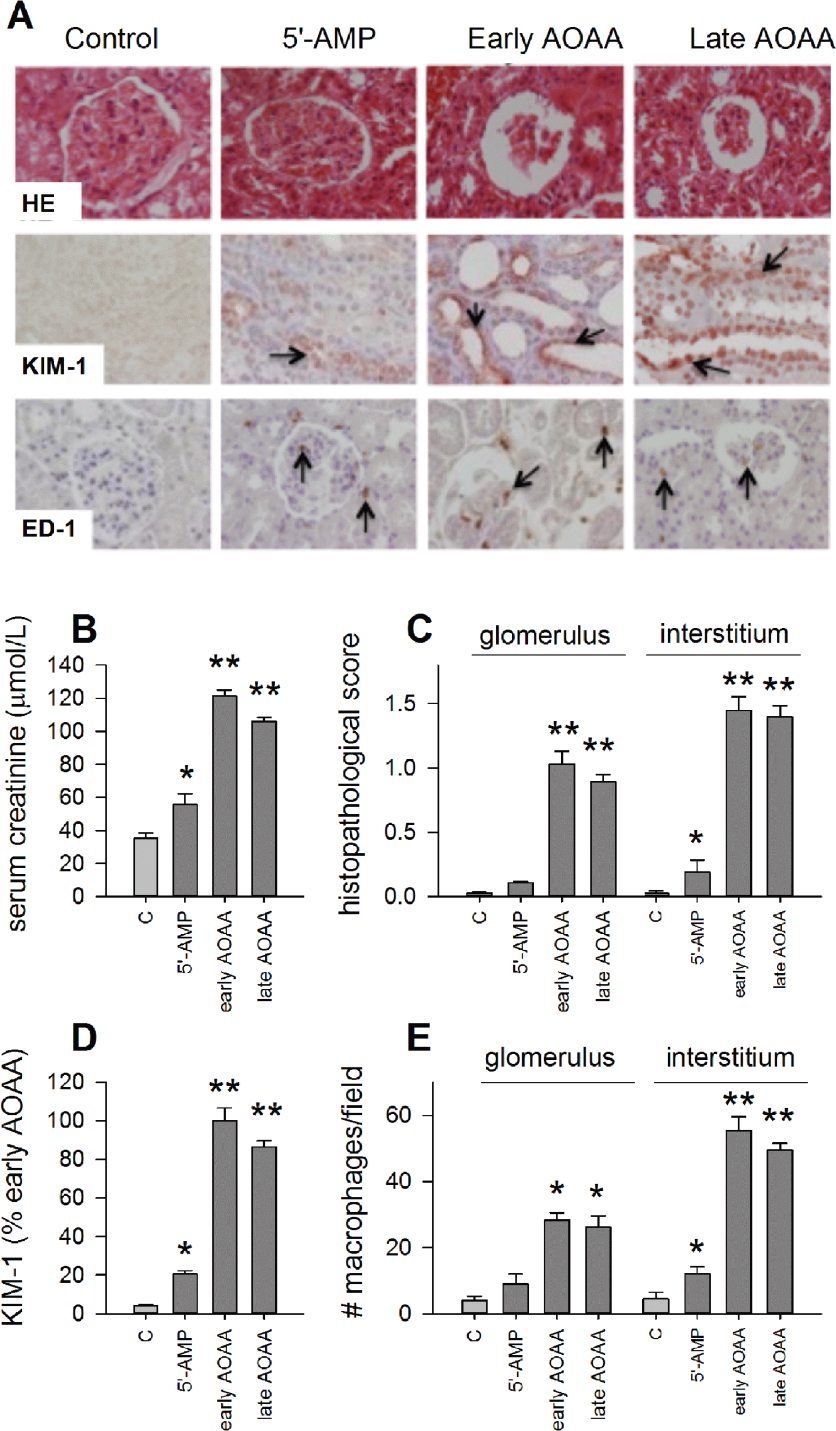
Blocking H_2_S production during 5’-AMP-induced torpor provokes kidney injury. (A) Representative photographs of kidney tissue; magnification x400. (B) Administration of 5’-AMP significantly increased plasma creatinine level compared to control animals, which was further elevated by AOAA injection prior to or 4 h after 5’-AMP administration. (C-E) Quantification of renal injury and markers, demonstrating modest renal injury in 5’-AMP induced torpor, which is grossly amplified by AOAA administration. HE = hematoxylin eosin staining; KIM-1 = Kidney Injury Molecule 1; ED-1 = antibody against CD68 specific for macrophages. */** represents *p < 0*.*05/0*.*01* compared to control. Arrows indicate positively stained areas.

In order to assess kidney function, we measured the plasma level of creatinine in all hamsters. During the torpor-like state induced by 5’-AMP, the level of creatinine in plasma is slightly increased as compared to control animals (Fig 2B; *p < 0*.*05*). Blocking endogenous H_2_S production with AOAA, either prior to the induction or during the torpor-like state, profoundly increased the plasma creatinine level, reaching levels around threefold higher as compared to control animals (Fig 2B; *p < 0*.*01*). Thus, induction of a torpor-like state by 5’-AMP leads to slightly elevated plasma creatinine level, which is augmented upon inhibition of endogenous H_2_S production. Potentially, H_2_S mediates preservation of the kidney function during the torpor-like state induced by 5’-AMP.

### Blocking H_2_S production is associated with glomerular and tubulointerstitial injury

Induction of torpor by 5’-AMP did not affect the morphology of glomeruli (Fig 2A and 2C *p > 0*.*05*), but was associated with minor signs of tubulointerstitial injury associated with influx of a low number of macrophages into the renal interstitium as compared to the control group (Fig 2A, 2C and 2E; *p < 0*.*05*). Further, injection of 5’-AMP resulted in a slight increase in the amount of KIM-1 protein in the renal tubules as compared to control group (Fig 2A and 2D; *p < 0*.*05*). To further substantiate the role of endogenous H_2_S production on renal morphology, renal sections from animals treated with AOAA were analyzed. Blocking endogenous H_2_S production with AOAA, either prior to or during 5’-AMP-induced torpor, enhanced glomerular, tubular and interstitial damage that was associated with a substantial influx of macrophages in the renal interstitium as compared to control animals (Fig 2A, 2C and 2E; *p < 0*.*01*). The higher level of renal injury during torpor is reflected by an increased amount of KIM-1 following blockade of H_2_S production (Fig 2A and 2D; *p < 0*.*01*). There was no significant difference in KIM-1 expression between early and late AOAA groups (Fig 2D; *p > 0*.*05*). Hence, 5’-AMP is associated with minor signs of tubulointerstitial injury. Although H_2_S is not essential for the induction of torpor, blockade of endogenous H_2_S production leads to pronounced glomerular and tubulointerstitial injury, thus suggesting a protective role of H_2_S against renal injury.

## Discussion

### H_2_S is not essential for the induction of a torpor-like state by 5’-AMP, but seems to play a key role in preserving kidney function and integrity

In the current study, we reveal that the induction of a torpor-like state by 5’-AMP in natural hibernators is not dependent on production of endogenous H_2_S. Blocking H_2_S production by AOAA, did not preclude torpor and did not induce an arousal. Remarkably, the torpor-like state induced by 5’-AMP is associated with increased plasma levels of H_2_S. The increased amount of all three H_2_S-producing enzymes by 5-AMP may account for the higher levels of H_2_S. Pharmacological induction of torpor by 5’-AMP leads to a slight increase in the plasma creatinine level and minor signs of tubulointerstitial injury, associated with a small influx of macrophages. Blocking endogenous H_2_S production with AOAA, either prior to or during 5’-AMP-induced torpor, enhanced glomerular, tubular and interstitial damage that was associated with a substantial influx of macrophages in the renal interstitium as compared to control animals [22]. Thus, in line with the role of endogenous H_2_S in preserving renal integrity during natural torpor and consistent with the renal protection during exogenously applied H_2_S in mouse [23–25], H_2_S seems to play a key role in mediating kidney preservation during pharmacologically induced torpor by 5’-AMP. However, H_2_S is not involved in the induction or maintenance of torpor induced by 5’-AMP.

### The mechanisms underlying 5'-AMP induction of torpor-like state remain to be unraveled

As described, our data demonstrate that H_2_S does not play an essential role in the induction of torpor by 5’-AMP. As an alternative explanation, activation of adenosine receptors, adenosine monophosphate protein kinase (AMPK) and adenylate kinase may lead to the induction of a torpor-like state. Swoap *et al*. [14] suggested that activation of adenosine receptors following dephosphorylation of 5'-AMP to adenosine may lead to lowering of the body temperature secondary to a reduction in cardiac output. This hypothesis is supported by the observation that not only (5’-)AMP, but also ATP, ADP and adenosine can induce a torpor-like state in mice and that lowering of the body temperature is blunted by co-treatment with an adenosine receptor antagonist [14]. The second hypothesis describes a role for AMPK, a key enzyme that plays a role in cellular energy homeostasis, which can be activated by depletion of cellular ATP (and consequently elevate AMP), and switches off energy consuming metabolic pathways [14,26–28]. Activation of signaling pathways downstream of AMPK promote a shift from anabolic towards catabolic processes and thereby reduce energy expenditure of the cells. However, it is unclear whether this leads to torpor-like behavior of the animal. Furthermore, activation of AMPK by intracerebroventricular infusion of AICAR (a specific AMPK-activator) in yellow-bellied marmots (*Marmota flaviventris)* during interbout arousal does not induce torpor, but lead to increased food intake and even prevents the return to torpor [29]. As a third hypothesis, relatively high levels of AMP lead to activation of adenylate kinase, which converts (5’-)AMP together with ATP to ADP. Injection of 5’-AMP may thereby lead to a relative ATP-depletion, which is implicated to reduce metabolism as observed during entrance into torpor [30]. Hence, the mechanism by which 5’-AMP induces a torpor-like state, and potentially natural torpor as well, remain to be unraveled. We reveal that pharmacological induction of a torpor-like state by 5’-AMP does not depend on H_2_S.

## Conclusion

Taken together, we demonstrate that 5’-AMP induces a torpor-like state in natural hibernators, leading to a lowering of the body temperature that is independent of the activation of H_2_S system. Although H_2_S does not seem to play an essential role in the induction of a torpor-likes state by 5’-AMP, endogenous production of H_2_S seems to play an essential role in precluding glomerular and tubulointerstitial renal injury and maintaining renal function. The exact mechanism(s) through which 5’-AMP induces a torpor-like state is not yet understood. Unraveling these molecular mechanisms may lead to the development of novel pharmacological therapies to safely reduce the metabolism to limit (hypothermic) IRI and thereby improve the outcome following organ transplantation and major cardiac/brain surgery.
